# Supplementary material for: Bacterial Carbonate Precipitation Using Active Metabolic Pathway to Repair Mortar Cracks
Source: Materials (Basel). 2022 Sep 23;15(19):6616. doi: 10.3390/ma15196616 (PMC9571396; doi:10.3390/ma15196616)
Supplement: Supplementary file 1 [file materials-15-06616-s001.zip › materials-1779431-supplementary.pdf]

Supplementary Materials

The following are the supplementary data related to this article.

Supplementary Materials S1

Image processing protocol.

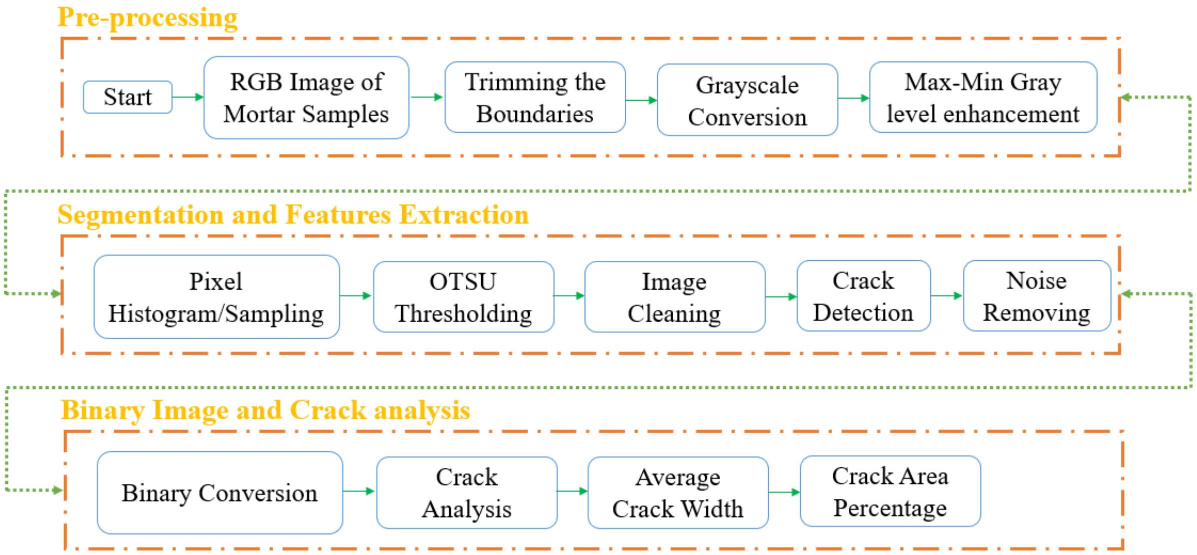

Supplementary Materials S2

Process of crack repair

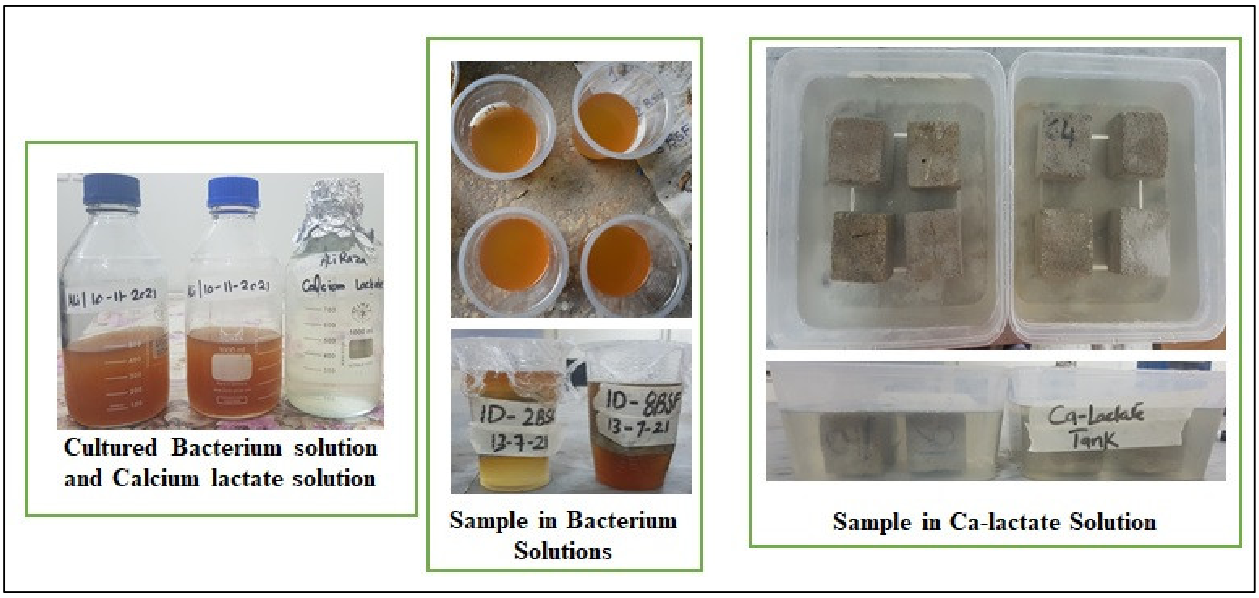

Supplementary Materials S3

Biodeposition repair without Ca-source (a-d), with Ca-source (e-i) and distilled water repair (j-l)

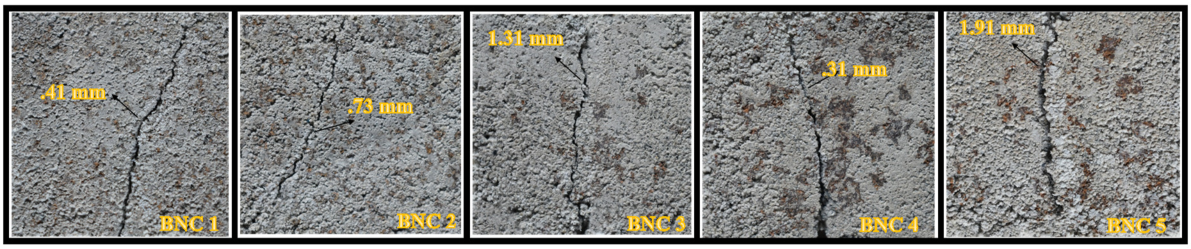

(a) Cracked mortar samples for **"Bac. Pum. No Ca"** formulation

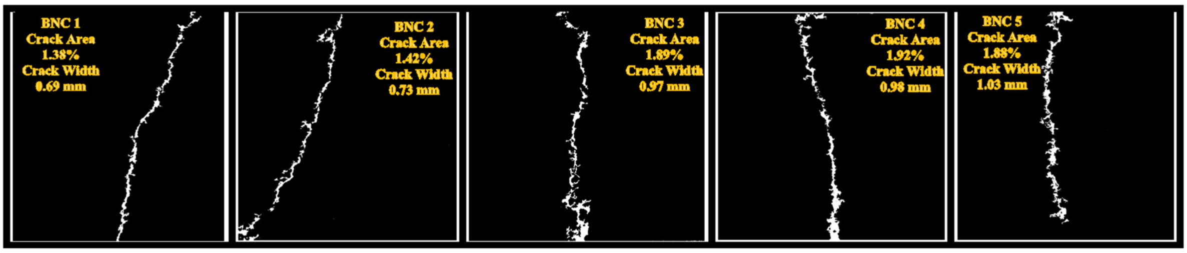

(b) Binary images of mortar samples for **"Bac. Pum. No Ca"** formulation (0 cycles)

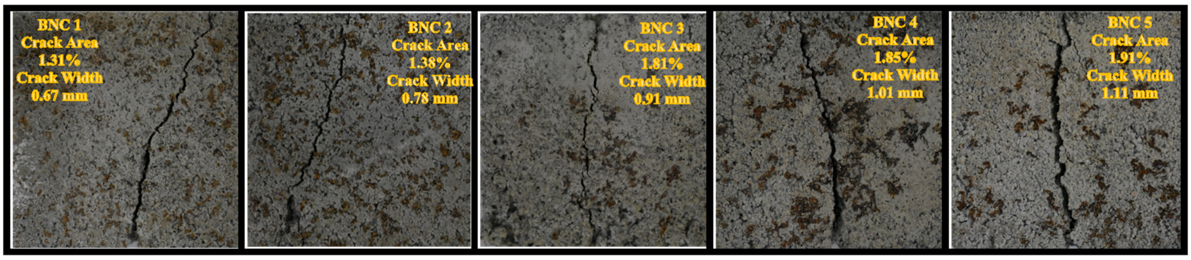

(c) After 7 cycles of **"Bac. Pum. No Ca"** repair treatment

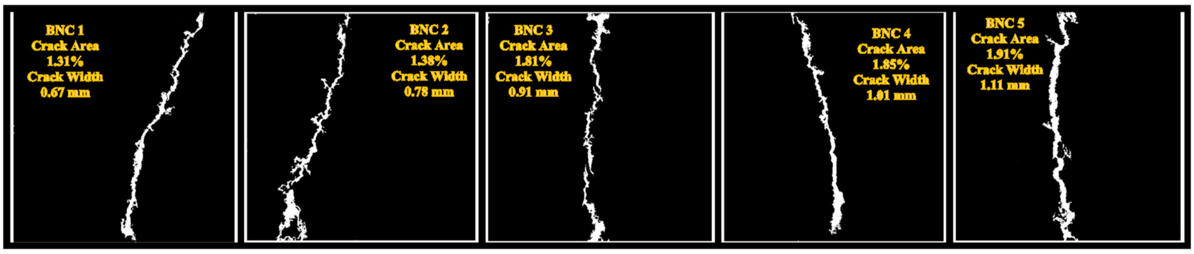

(d) Binary image after 7 cycles of **"Bac. Pum. No Ca"** repair treatment

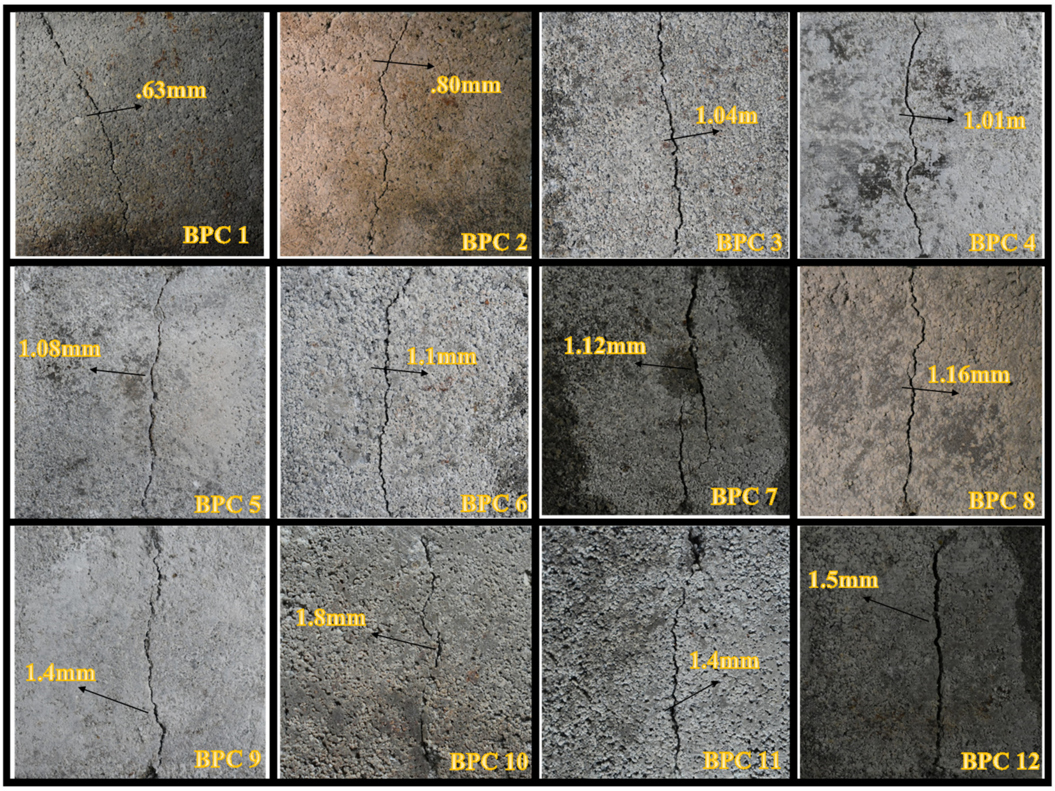

(e) Cracked mortar samples for **"Bac. Pum. Ca"** formulation

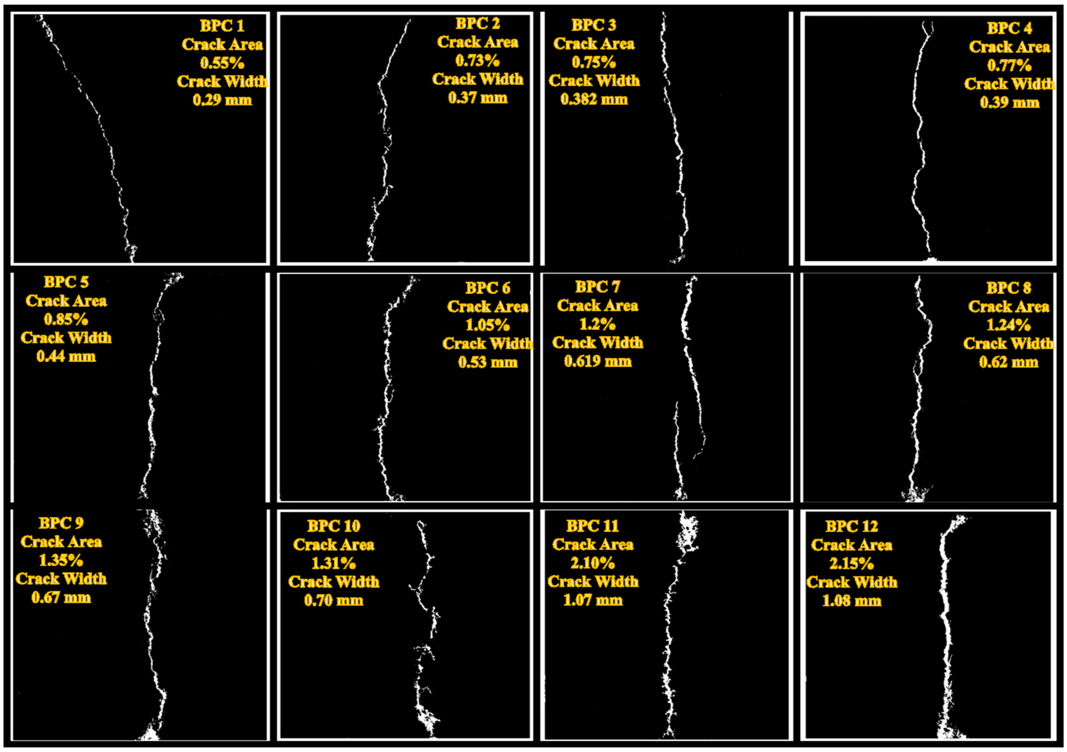

(f) Binary images of mortar samples for **"Bac. Pum. Ca"** formulation (0 cycles)

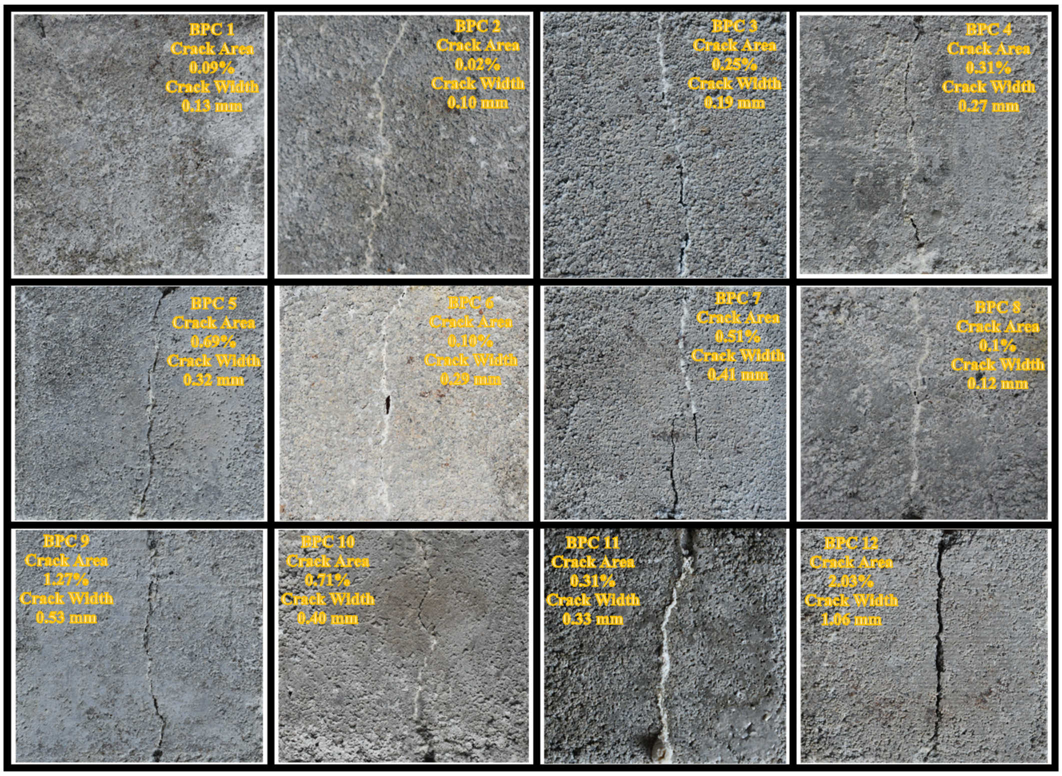

(g) After 7 cycles of **"Bac. Pum. Ca"** repair treatment

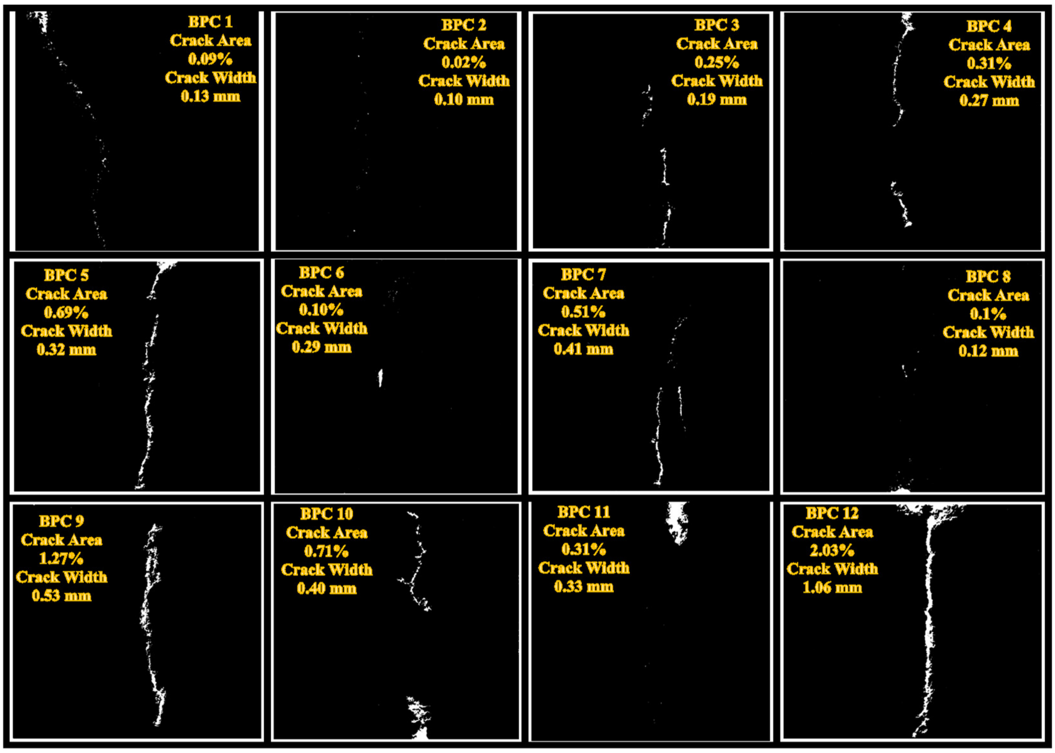

(h) Binary image of samples repaired with **"Bac. Pum. Ca"** formulation (7 cycles)

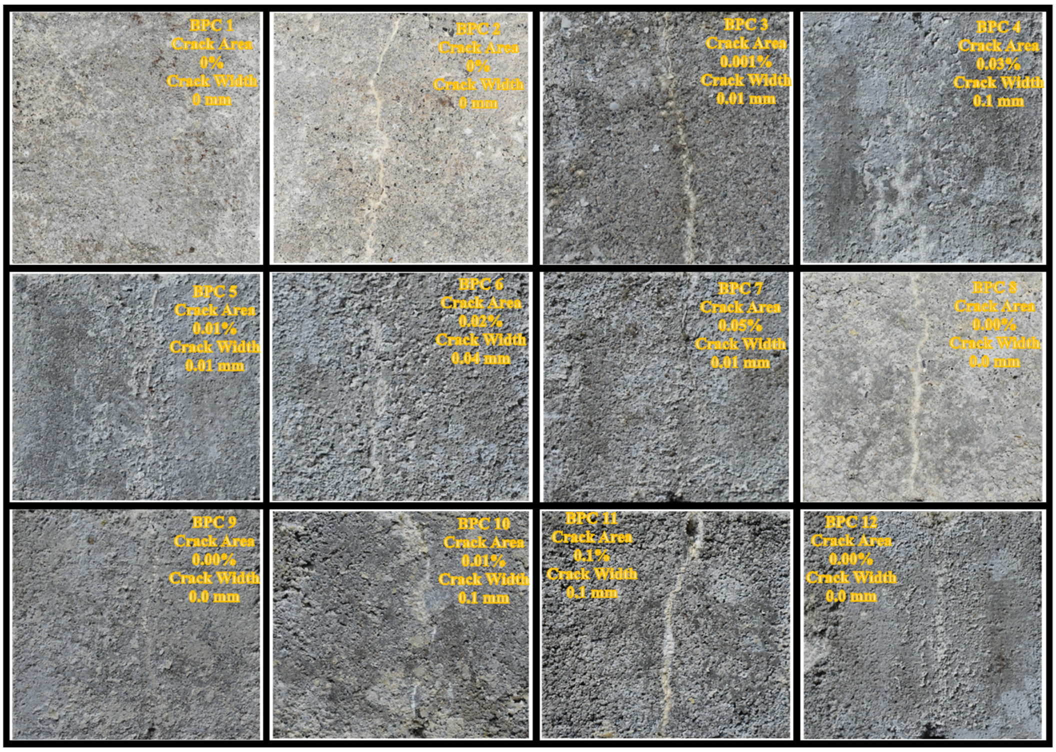

(i) After 14 cycles of **"Bac. Pum. Ca"** repair treatment

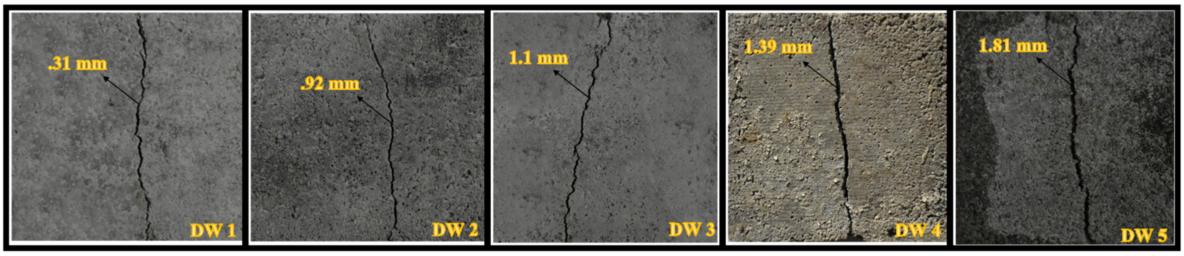

(j) Cracked mortar samples for **"DW"** formulation

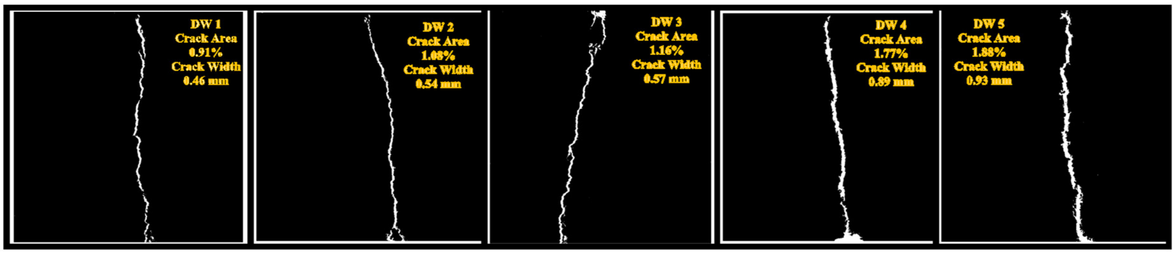

(k) Binary images of mortar samples for **"DW"** formulation (0 cycles)

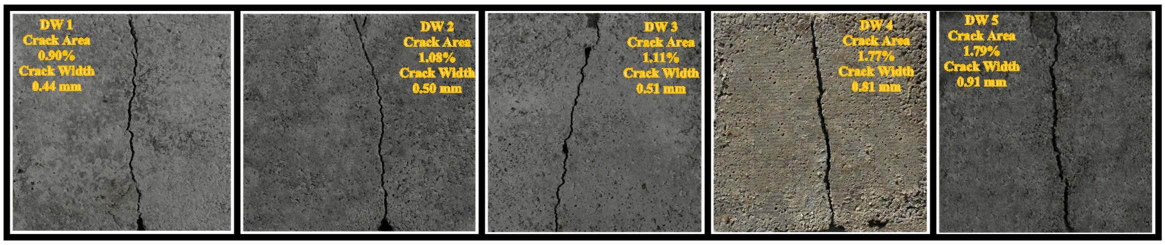

(l) Mortar samples after 7 cycles of **"DW"** repair treatment

Author Contributions S4

Crack features with test results of different repairs

Type of Repair	ID	Initial Crack		Water Permeability (m/s)			Healed Area (%)		Water Tightness (%)		Compressive Strength	
		Ave. width (mm)	Crack Area (%)	0 Cycle	7 Cycles	14 Cycles	7 Cycles	14 Cycles	7 Cycles	14 Cycles	Repaired Compressive Strength N/mm^2	Compressive Strength Recovered (CSR) (%)
**Biodeposition with Calcium lactate** Bac. Pum. Ca	**BPC1**	0.29	0.55	9.4603E-07	9.2995E-07	7.4396E-07	83.64	100.00	1.70	21.36	**31.88**	**74.80**
	**BPC2**	0.37	0.73	1.5839E-06	9.9752E-07	9.5875E-07	97.26	100.00	37.02	39.47	**33.4**	**78.37**
	**BPC3**	0.382	0.75	1.8855E-06	1.5291E-06	9.3632E-07	66.67	99.87	18.90	50.34	**26.12**	**61.29**
	**BPC4**	0.39	0.77	2.2784E-06	1.8855E-06	1.6162E-06	59.74	96.10	17.24	29.06	**29.68**	**69.64**
	**BPC5**	0.44	0.85	2.5141E-06	2.3983E-06	9.5596E-07	18.82	98.82	4.61	61.98	**29.72**	**69.73**
	**BPC6**	0.53	1.05	3.2324E-06	2.0712E-06	1.9812E-06	90.48	98.10	35.92	38.71	**27.28**	**64.01**
	**BPC7**	0.619	1.20	1.0285E-05	9.9928E-06	4.5567E-06	57.50	95.83	2.84	55.69	**28.52**	**66.92**
	**BPC8**	0.62	1.24	1.3576E-05	9.0506E-06	7.2989E-06	91.94	100.00	33.33	46.24	**32.84**	**77.06**
	**BPC9**	0.67	1.35	1.5189E-05	1.1991E-05	7.5946E-06	5.93	100.00	21.05	50.00	**33.72**	**79.12**
	**BPC10**	0.7	1.31	2.2784E-05	1.0131E-05	8.7025E-06	45.80	99.24	55.53	61.80	**29.24**	**68.61**
	**BPC11**	1.07	2.10	1.3670E-04	1.1392E-05	7.5946E-06	85.24	95.24	91.67	94.44	**30.48**	**71.52**
	**BPC12**	1.08	2.15	1.8227E-04	1.2531E-04	9.3953E-06	5.58	100.00	31.25	94.85	**32.52**	**76.30**
**Biodeposition without Calcium lactate** Bac. Pum. No Ca	**ID**	**Ave. width (mm)**	**Crack Area (%)**	**0 Cycle**	**5 Cycles**	**7 Cycles**	**5 Cycles**	**7 Cycles**	**5 Cycles**	**7 Cycles**	**Repaired Compressive Strength N/mm^2**	**Compressive Strength Recovered (CSR) (%)**
	**BNC1**	0.69	1.38	1.0315E-05	1.0131E-05	1.0202E-05	3.62	5.07	1.78	1.10	20.92	49.09
	**BNC2**	0.73	1.42	1.6275E-05	1.4546E-05	1.4647E-05	0.70	2.82	10.63	10.01	17.16	40.27
	**BNC3**	0.97	1.89	1.0465E-04	7.9193E-05	7.9743E-05	2.12	4.23	24.32	23.80	23.72	55.66
	**BNC4**	0.98	1.92	1.1313E-04	1.0182E-04	1.0253E-04	1.04	3.65	10.00	9.37	19.92	46.74
	**BNC5**	1.03	1.88	1.0182E-04	1.0079E-04	1.0356E-04	0.53	1.60	1.01	-1.71	19.32	45.34
Distilled Water Dist. Wat	**DW1**	0.46	0.91	9.05E-06	8.54E-06	7.67E-06	1.10	1.10	5.66	15.25	23.32	54.72
	**DW2**	0.54	1.08	9.63E-06	9.32E-06	8.44E-06	6.48	8.33	3.24	12.37	24.08	56.50
	**DW3**	0.57	1.16	1.05E-05	9.7E-06	7.94E-06	0.86	4.31	7.86	24.56	21.28	49.93
	**DW4**	0.89	1.77	1.13E-05	9.49E-06	8.7E-06	3.39	3.95	16.09	23.08	20.44	47.96
	**DW5**	0.93	1.88	1.51E-05	1.47E-05	1.38E-05	4.26	4.79	2.55	8.46	22.48	52.75
	**Min Value**	0.29	0.55	9.4603E-07	9.2995E-07	7.4396E-07	5.58	95.24	1.70	21.36	**26.12**	**61.29**
	**Max Value**	1.08	2.15	1.8227E-04	1.2531E-04	9.3953E-06	97.26	100.00	91.67	94.85	**33.72**	**79.12**

Author Contributions S5

Ultra-sonic results of different repairs

Type of Repair	ID	Initial Crack		Ultrasonic Pulse Velocity (m/s)					
		Ave. width (mm)	Crack Area (%)	Before crack	0 cycle	5 cycles	7 cycles	12 cycles	14 cycles
Biodeposition with Calcium lactate Bac. Pum. Ca	BPC1	0.29	0.55	4456.14	3078.79	3577.46	3628.57	3877.86	3907.69
	BPC2	0.37	0.73	4341.88	2970.76	3298.70	3409.40	3762.96	3877.86
	BPC3	0.382	0.75	4268.91	2673.68	2970.76	3023.81	3320.26	3762.96
	BPC4	0.39	0.77	4618.18	2659.69	2673.68	2953.49	3277.42	3602.84
	BPC5	0.44	0.85	3907.69	2490.20	2687.83	2853.93	3155.28	3342.11
	BPC6	0.53	1.05	4233.33	2454.11	2806.63	2902.86	3432.43	3654.68
	BPC7	0.619	1.20	4268.91	2288.29	2362.79	2552.76	3135.80	3342.11
	BPC8	0.62	1.24	4198.35	2298.64	2527.36	2716.58	3277.42	3602.84
	BPC9	0.67	1.35	4064.00	2288.29	2351.85	2605.13	2970.76	3552.45
	BPC10	0.7	1.31	4341.88	2267.86	2341.01	2540.00	2775.96	2953.49
	BPC11	1.07	2.10	4417.39	2143.46	2396.23	2540.00	2659.69	3256.41
	BPC12	1.08	2.15	4130.08	2116.67	2161.70	2309.09	2540.00	2902.86
Biodeposition without Calcium lactate Bac. Pum. No Ca	BNC1	0.69	1.38	3877.86	2319.63	2341.01	2384.98	--	--
	BNC2	0.73	1.42	3968.75	2218.34	2228.07	2116.67	--	--
	BNC3	0.97	1.89	3968.75	2107.88	2116.67	2161.70	--	--
	BNC4	0.98	1.92	4198.35	2116.67	2134.45	2180.26	--	--
	BNC5	1.03	1.88	3907.69	1976.65	2000.00	2007.91	--	--
Distilled Water Dist. Wat	DW1	0.46	0.91	4198.35	2527.36	2514.85	2478.05	--	--
	DW2	0.54	1.08	3937.98	2407.58	2419.05	2384.98	--	--
	DW3	0.57	1.16	4268.91	2341.01	2351.85	2341.01	--	--
	DW4	0.89	1.77	3791.04	2228.07	2228.07	2237.89	--	--
	DW5	0.93	1.88	3937.98	2134.45	2143.46	2143.46	--	--
